# Structural and enzyme kinetic studies of retrograded starch: Inhibition of α-amylase and consequences for intestinal digestion of starch

**DOI:** 10.1016/j.carbpol.2017.01.040

**Published:** 2017-05-15

**Authors:** Hamung Patel, Paul G. Royall, Simon Gaisford, Gareth R. Williams, Cathrina H. Edwards, Frederick J. Warren, Bernadine M. Flanagan, Peter R. Ellis, Peter J. Butterworth

**Affiliations:** a King’s College London, Faculty of Life Sciences and Medicine, Diabetes and Nutritional Sciences Division, Biopolymers Group, Franklin-Wilkins Building, 150 Stamford Street, London SE1 9NH, United Kingdom; b King’s College London, Faculty of Life Sciences and Medicine, Institute of Pharmaceutical Science, Drug Delivery Group, Franklin-Wilkins Building, 150 Stamford Street, London SE1 9NH, United Kingdom; c UCL School of Pharmacy, University College, 29-39 Brunswick Square, London WC1N 1AX, United Kingdom; d Institute of Food Research, Norwich Research Park, Colney, Norwich NR4 7UA, United Kingdom; e Excellence in Plant Cell Walls, Centre for Nutrition and Food Sciences, Queensland Alliance for Agriculture and Food Innovation, University of Queensland, St. Lucia, Brisbane, QLD 4072, Australia

**Keywords:** GLUT2, plasma membrane glucose transporter, HAMS, high amylose maize starch, RHAMS, retrograded high amylose maize starch, SGLT, plasma membrane sodium dependent glucose transporters, Starch retrogradation, FTIR-ATR, MC-DSC, XRD, Amylase inhibition

## Abstract

Retrograded starch is known to be resistant to digestion. We used enzyme kinetic experiments to examine how retrogradation of starch affects amylolysis catalysed by porcine pancreatic amylase. Parallel studies employing differential scanning calorimetry, infra red spectroscopy, X-ray diffraction and NMR spectroscopy were performed to monitor changes in supramolecular structure of gelatinised starch as it becomes retrograded. The total digestible starch and the catalytic efficiency of amylase were both decreased with increasing evidence of retrogradation. A purified sample of retrograded high amylose starch inhibited amylase directly. These new findings demonstrate that amylase binds to retrograded starch. Therefore consumption of retrograded starch may not only be beneficial to health through depletion of total digestible starch, and therefore the metabolisable energy, but may also slow the rate of intestinal digestion through direct inhibition of α-amylase. Such physiological effects have important implications for the prevention and management of type 2 diabetes and cardiovascular disease.

## Highlights

• Amylase catalytic efficiency and starch digestibility decrease as starch retrogrades.
• Retrograded starch binds to amylase and inhibits catalytic activity.
• Amylase inhibition has important implications for slowly digestible starch design.

## 1 Introduction

Starch is a major energy-providing carbohydrate in the human diet and is also used in a number of industrial applications that have become of considerable economic importance (Antoni, Zverlov & Schwarz, 2007; Warren, Butterworth & Ellis, 2013). Such applications include alcohol production from the fermentation of starch and bioethanol production from enzymically hydrolysed maize starch.

In the human diet, the main source of digestible starch is that present in foods derived from cereal crops such as wheat, rice and maize, from root crops including potato and yams and also from legumes such as beans and peas. The first stage in the digestion of starch involves hydrolysis catalysed by salivary and pancreatic α-amylases to produce maltose, maltotriose and α-limit dextrins predominantly (Roberts & Whelan, 1960). These sugars are then hydrolysed to glucose by the brush border disaccharidases maltase-glucoamylase and sucrase-isomaltase (Nichols et al., 2003, Sim et al., 2010). Glucose is then absorbed from the intestinal mucosa into the portal blood through GLUT2 and SGLT transporters (Kellet & Brot-Laroche, 2005). Digestion of starch-rich foods is followed by postprandial rises in blood glucose and insulin concentrations, but the postprandial response to foods with similar starch contents can differ greatly. The glucose response of foods is frequently described by their glycaemic index (GI) value (Jenkins et al., 2002) and many studies have linked low GI diets with reduced risk of developing type 2 diabetes mellitus and cardiovascular disease (Jenkins et al., 2002, Wolever et al., 1992). Any remaining undigested starch eventually reaches the colon where it is metabolised by microflora to short chain fatty acids such as propionate and butyrate. These metabolites play an important role in maintenance of colonic epithelial cells and in protection against intestinal disease (Canani, Di Constanzo, Leone, Pefata, & Calignano, 2011; Cummings & Branch, 1986).

The variations in glycaemic responses are mainly attributed to differences in the rate and extent of amylolysis of different starches (Goni, Garcia-Alonso, & Saura-Calixto, 1997). The supramolecular structure of starch that is affected by starch processing (e.g., hydrothermal treatment) together with the bioaccessibility of starch contained within a food matrix are significant factors in influencing amylolysis (Edwards et al., 2015; Liu, Donner, Yin, Huang & Fan, 2006). Many food processing techniques involve hydrothermal treatment followed by cooling, which cause structural changes at the granular and molecular level. Different processing conditions and botanical sources can greatly affect starch structure and availability, with implications for starch digestibility. Heating native starch (50–100 °C) in excess water (e.g. 1%, w/w, aqueous starch dispersion) leads to an endothermic gelatinisation. This is characterised by an ordered to disordered transition as a result of physical changes of starch including granule swelling and leaching of mainly soluble amylose. During this process the crystallites begin to melt and the semi-crystalline starch structure is completely destroyed, at which point the disruption of the original structure becomes irreversible (Htoon et al., 2009). Starches from different botanical sources differ in their gelatinisation behaviour, and not all starches are completely gelatinised during hydrothermal processing, especially when heated at low water contents (<30%, w/w, water in starch solutions) or in low moisture foods (Roder et al., 2009). Previous work shows that complete gelatinisation of high amylose mutant starches is only achieved at temperatures in excess of 120 °C (Haralampu, 2000, Jacobs and Delcour, 1998; Sajilata, Singhal & Kulkarni, 2006; Sievert & Pomeranz, 1989; Tahir, Ellis & Butterworth, 2010).

When gelatinised starch is stored it begins to acquire a more ordered structure upon cooling and becomes resistant to α-amylase attack. This phenomenon is termed retrogradation (Htoon et al., 2009). Retrogradation involves the formation of a gel-like texture with association of amylose chains and double helix formation and by association of amylopectin chains into double helices. Retrogradation of amylose chains occurs at a much faster rate than those of amylopectin (Haralampu, 2000). Retrogradation is commonly observed in the staling of baked foods such as bread and biscuits and involves the association of the amylopectin chains (Hug-Iten, Escher & Conde-Petit, 2003). It has been known for some time that retrograded starch is resistant to amylase digestion both *in vitro* and *in vivo* (Englyst & Cummings, 1985).

Given the differences in the rates at which food starch is digested and the desirability for human health of diets containing starch-rich low GI foods that are digested relatively slowly, studies of structure and physicochemical properties of starch have attempted to elucidate key factors that affect the rate at which starch is hydrolysed by α-amylase (Butterworth, Warren & Ellis, 2011; Mahasukhonthat, Sopade & Gidley, 2010). Previously we described an *in vitro* mechanistic approach for studying how retrograded starch affects amylolysis catalysed by porcine pancreatic amylase (PPA) (Patel, Day, Butterworth & Ellis, 2014). We now report further investigation of this subject area. A novel enzyme kinetic study on *in vitro* amylolysis combined with structural analysis of retrograded starch was undertaken. In addition, we show for the first time, that not only is retrograded starch inert to PPA catalysed hydrolysis during the normal time course of digestion, it can also have a direct inhibitory effect on amylase action.

## 2 Materials and methods

### 2.1 Starches and chemicals

Wheat (Cerestar, CV.GL.04) and pea starches were gifts from Prof. C. Hedley and Prof. T. Bogracheva (formerly of the John Innes Centre, Norwich, UK). Potato starch was obtained from the National Starch and Chemical Company (member of the ICI group, London, UK) and starches from maize, waxy maize and high amylose maize (Gelose 80) were gifts from Dr. C. Pelkman, Ingredion UK (Manchester, Lancashire, UK).

Phosphate buffered saline (PBS) tablets were purchased from Oxoid Ltd. (Basingstoke, Hampshire, UK) and PPA (type 1A DFP treated) was obtained from Sigma-Aldrich Company Ltd. (Poole, Dorset, UK) and checked for purity by SDS-PAGE. The suppliers quoted activity of 1333 units per mg of protein is equivalent to 1293 IU per mg at 25 °C (Tahir et al., 2010)

### 2.2 Chemical composition and swelling of raw starches

Starch moisture was determined gravimetrically by weighing 100 mg of the starch sample onto pre–dried aluminium pans and heating at approximately 103 °C overnight. The dried weight was recorded and the moisture content was calculated by difference. The amylose/amylopectin content for the starches was analysed by iodine dye binding (Knutson, 1986, Knutson, 2000) and the protein content was determined using the bicinchoninic acid assay (Patel et al., 2014). For water uptake determination, weighed samples of approximately 100 mg of starch were gelatinised in 10 ml water at 60 °C for 30 min. Each sample was then centrifuged at 2500 × *g* for 15 min before the supernatant was discarded and the weight recorded (Daramola & Osanyinlusi, 2006).

### 2.3 Preparation and processing of starch

Stock suspensions (10 mg/ml) of all native starches listed above were prepared in PBS and agitated in a glass beaker on a magnetic hotplate for 20 min at room temperature (∼22 °C). Aliquots from the stock suspensions were then diluted into separate 15 ml Falcon™ tubes with PBS to provide the desired range of 0.5–10 mg/ml final concentrations of starch in a volume of 4 ml.

Gelatinised starch was prepared by heating sealed flasks containing 10 mg/ml stock suspensions in water at 90 °C. A thermocouple probe was used to monitor the temperature of the heating bath. Once the target temperature was reached the suspension was held for 20 min to allow for complete gelatinisation. All starches were treated in this way except high amylose maize which was pre-gelatinised at 100 °C for 10 min before autoclaving at 121 °C for 20 min to complete the gelatinisation. The pre-gelatinisation step ensures that starch sedimentation does not occur during autoclaving. All gelatinised starch suspensions were weighed before and after heating to check for loss of water by evaporation.

For retrograded starch, 10 mg/ml suspensions in distilled water were gelatinised as described above, and then 10–12 ml aliquots were placed into 15 ml Falcon™ tubes and set aside at room temperature for various periods of time from 6 to 96 h. At the end of the storage times, suitable volumes of the samples were diluted to 4 ml with PBS to achieve a range of concentrations required for further experiments. Because the rate of retrogradation can be concentration dependent (Eerlingen, Crombez & Delcour, 1993; Longton and Legrys, 1981, Zeleznak and Hoseney, 1986), appropriate dilutions were made at the end of each storage time to ensure that starch concentrations were the same for each sample being set aside for the different times. To study retrogradation at 4 °C, 10 mg/ml suspensions of gelatinised wheat and potato starches were stored in a cold room for 7 days. Sodium azide (0.01%, w/w) was added to the suspensions to inhibit microbial growth. The azide had no effect on amylase activity.

### 2.4 FTIR-ATR spectroscopy

FTIR-ATR spectra were obtained for native starches using a Perkin-Elmer Spectrum One^®^ spectroscope equipped with a SensIR technologies IR II Durascope^®^ diamond cell attenuated reflectance (ATR) device with a diamond crystal providing an incidence angle of 45° (Patel et al., 2014, Warren, Perston et al., 2013). Scanning was performed at room temperature (∼22 °C) over a wavelength range of 4000–550 cm^−1^. A total of 24 scans were co-added with a resolution of 4 cm^−1^. A background spectrum was obtained for water under the same conditions and subtracted from the starch spectrum prior to analysis. For gelatinised starches, 10 mg/ml suspensions in water were heated in a water bath at 90 °C for 20 min then cooled swiftly to room temperature before the spectra were taken. For retrograded material, gelatinised starch was stored at room temperature for up to 96 h with spectra determined every 24 h. Again, a background spectrum was performed for each new run. The amplitudes of absorbance at 1000 cm^−1^ and 1022 cm^−1^ were recorded for all spectra.

### 2.5 Differential scanning calorimetry (DSC)

DSC thermograms were obtained for a number of starches including high amylose maize (native and retrograded) using a TA instruments Multi-Cell Differential Scanning Calorimeter (MC DSC). Weighed samples of native starch (approximately 50 mg) in 1 ml of water were placed in steel ampoules and heated at a rate of 0.5 °C/min (Bogracheva, Wang, Wang & Hedley, 2002) while scanning from 20 to 100 °C, or to 150 °C in the case of high amylose maize to ensure complete gelatinisation when using this starch. An ampoule containing water was used as a reference. The samples were then cooled to 20 °C at a rate of 1 °C per min. The instrument chamber was constantly purged with nitrogen at a flow rate of 50 ml per min throughout the experiments. Gelatinisation temperatures including those for the onset (T_o_), peak (T_p_) and conclusion (T_c_) were obtained for all starches. For gelatinised starches, DSC was used to examine each sample after a cooling period at 20 °C of 30 min only, so as to minimise retrogradation. For retrograded material, two starch suspensions were prepared namely, 50 mg/ml and 200 mg/ml of distilled water. The 50 mg/ml samples were gelatinised by heating in the DSC instrument and then the ampoules containing these samples were stored at room temperature for 48 h to produce retrograded material. This retrograded starch was then heated from 20 to 150 °C at a rate of 0.5 °C per min and thermograms recorded. Comparable experiments were performed with starch samples of 200 mg per g water in order to obtain larger heat flow signals in the steel ampoules, but after gelatinisation, the ampoules were stored for 1 week at 4 °C before being re-scanned.

### 2.6 Powder X-ray diffraction (XRD)

X-ray diffraction patterns were recorded for high amylose maize starch (HAMS) and retrograded high amylose maize starch (RHAMS) using a Rigaku Miniflex 600 instrument with CuKα radiation (λ = 1.548 Å) at 40 kV and 15 mA. A small quantity of starch powder was mounted on an aluminium sample holder and levelled with a glass slide. The samples were scanned over an angular range (2Ɵ) of 3–40° and analysed using OriginPro 9.1^©^ software (available online at http://www.originlab.com).

### 2.7 ^13^C CP/MAS.NMR spectroscopy

Samples of native and retrograded HAMS were examined in the solid state by ^13^C CP/MAS.NMR spectroscopy (Gidley & Bociek, 1985) and the total helical content was calculated using a published predictive fitting method (Flanagan, Gidley &Warren, 2015).

### 2.8 Preparation of retrograded high amylose maize starch (purified RHAMS)

A suspension in distilled water of high amylose maize starch (10 mg/ml) was gelatinised at 100 °C for 10 min prior to autoclaving at 121 °C for 20 min. The treated samples were stored at 4 °C for 24 h followed by storage at 37 °C for another 24 h (Park, Baik & Lim, 2009). This 4 °C/37 °C cycle was repeated three more times. Any remaining non-retrograded material was eliminated by digestion with amylase and amyloglucosidase. To each Falcon™ tube, 3 ml of PPA (10 mg/ml) containing amyloglucosidase (3 U/ml) (Megazyme International, Bray, County Wicklow, Ireland) was added and vortex mixed. All tubes were then placed on a rotary table that provided end-over-end mixing in an incubator at 37 °C for 18 h. The reaction was terminated by adding ethanol and samples were centrifuged to sediment the starch. The supernatant was discarded and the paste material was freeze-dried to a powder form. A Resistant Starch Kit, Megazyme AOAC 2002.02 Official Method. (Megazyme International, Bray, County Wicklow, Ireland) was used to estimate the concentration of retrograded starch in the RHAMS preparation and for the digestion with amylase plus amyloglucosidase reagent described above.

To check the quality of the purified RHAMS, the chemical composition was determined as described above for the raw starches. The mean values (w/w) ± s.e.m. obtained for the amylose, water and protein contents of RHAMS were 94.2 ± 1.1%, 3.5. ± 0.3% and 0.65 ± 0.03% respectively. The low water content was expected because of freeze drying during the preparation.

### 2.9 Initial rates of digestion with α-amylase and kinetic data analysis

The method was essentially that described in detail previously (Slaughter, Ellis & Butterworth, 2001). Tubes with a range of starch concentrations of 0.5–10 mg/ml in PBS were equilibrated at 37 °C for up to 20 min with constant mixing. The temperature was monitored with a digital thermocouple. The digestion was initiated by adding amylase to a final concentration of 1.2 nM. Replicate 300 μl aliquots were removed at incubation times of 0, 4, 8 and 12 min and added to Eppendorf tubes containing 300 μl of ice cold 0.3 M Na_2_CO_3_ solution to quench the reaction. After centrifugation and collection of the supernatant, the total reducing sugar released during amylolysis was assayed by a Prussian blue method (Slaughter et al., 2001) and expressed as maltose equivalents by comparison with a standard curve of 0–100 μM produced for this sugar. Samples from the enzyme incubation mixtures were diluted suitably before assay to bring them within the range of the standard curve.

Initial rates of digestion were calculated from the slopes of plots of maltose concentration against time and used for determination, by regression, of the values of *K*_m_, *V*_max_ and then *k*_cat_ (from V_max_/[PPA]). Non-linear regression fits to the Michaelis-Menten equation was undertaken using Sigmaplot 12^©^ software. Catalytic efficiencies (CE) were obtained from *k*_cat_/*K*_m_ values.

### 2.10 Inhibition of amylolysis by RHAMS

A 10 mg/ml suspension of RHAMS was prepared in PBS and agitated gently for 10 min by swirling. This was diluted in PBS to give a working inhibitor concentration of 2.5 mg/ml. To achieve the concentrations of inhibitor used in the experiments, appropriate aliquots of the working suspension were added to 15 ml Falcon™ tubes containing gelatinised wheat starch at various concentrations ranging from 0.5 to 2.0 mg/ml. Inhibitor (RHAMS) concentrations varied between 0 and 1 mg/ml. The final volume of each reaction mixture was 4 ml. Amylolysis under the conditions already described was then initiated by an addition of PPA (final concentration 1.2 nM) and samples taken for assay of reducing sugar content and determinations of initial rates of reaction.

### 2.11 Digestibility constants and total digestible starch (C_∞_)

Digestibility assays at 37 °C for native starch samples of potato, wheat, pea, maize, waxy maize starches and HAMS, were performed using suspensions containing 5 mg/ml starch and 4.5 nM PPA. Aliquots were removed at various time points up to 120 min for determination of reducing sugars. Similar experiments were conducted with gelatinised starches and 24 h retrograded starch, but with the addition of 2.25 nM PPA. Digestibility curves were analysed using the log of slope (LOS) application to 1st order kinetics (Butterworth, Warren, Grassby, Patel & Ellis, 2012) to obtain values for the digestibility rate constant, *k*, and the total amount of digestible starch, C_∞_.

## 3 Results and discussion

The various starches used in the study did not differ greatly in their protein and moisture content with values (w/w) ranging from 0.1 to 0.5% and 11 to 16%, respectively. The water content of RHAMS that had been freeze dried during purification was 3.5%. The amylose content ranged between 20 and 30% (w/w) for all starches except waxy maize (1.2% amylose) and our sample of HAMS (79.1% amylose). The water uptake potential was also tested for all starches and ranged from 6.6 to 10.0% (w/w) with only potato showing a relatively high value (20.6%), which can be mainly attributed to the high levels of phosphate ester groups attached to the glucose residues of the amylopectin fraction in potato starch (Slaughter et al., 2001).

The endothermic transition temperatures of native starches determined by DSC are shown in Supplementary Information (Table 1S). HAMS had the largest gelatinisation range (36 °C) and the lowest gelatinisation enthalpy (Δ*H*_gel_ of 5.5 J/g). For all the other starches, apart from potato (Δ*H*_gel_ of 17.5 J/g), enthalpy values were in the range of 9–12 J/g that agreed well with previous findings from our laboratory (Warren, Butterworth et al., 2013).

**Fig. 1:**
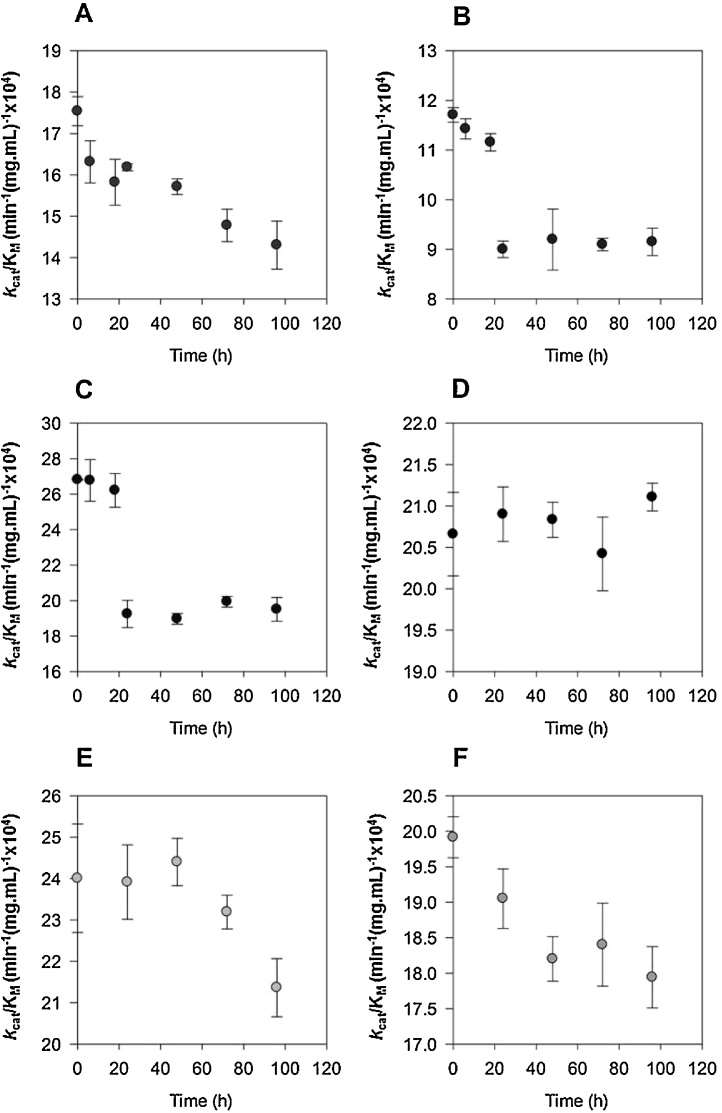
Effect of storage at 22 °C on the Catalytic Efficiency (k_cat_/*K*_m_) of PPA acting on various gelatinised starches. A, Wheat; B, HAMS, C; Potato, D; Waxy maize; E, Pea; F, Maize. All data points represent mean values ± s.e.m. from 3 to 4 replicate experiments.

Fig. 1 shows the change in catalytic efficiency (CE) of PPA action on the various starches with time of storage after gelatinisation. It is notable that the CE for gelatinised waxy maize starch changes little on storage in contrast to the other starches, which all show a decrease in CE with storage time. For potato and HAMS the decrease of CE is 20–25% within 20 h but the rate of change for the other starches is somewhat slower, particularly so for pea starch. When wheat and potato starches were stored at 4 °C for 7 days, the CE of both decreased further to a value that was approximately 60% of the fresh material (data not shown). The absence of a change in CE for waxy maize accompanying storage can probably be attributed to the known slow rate of amylopectin retrogradation (Haralampu, 2000) with the result that little retrograded amylopectin formed in the time period used in our experiments.

FTIR-ATR spectra have been usefully employed for examining the surface structure of starch granules and peaks at 1000 cm^−1^ and 1022 cm^−1^ are characteristic of ordered and amorphous regions of starch respectively (Capron, Robert, Colonna, Brogly & Planchot, 2007; Patel et al., 2014; Sevenou, Hill, Farhat, & Mitchell, 2002; Warren, Perston et al., 2013). The peak ratio of 1000 cm^−1^/1022 cm^−1^ can be a useful indicator of the proportion of ordered to disordered α-glucan chains at the exposed surface of the starch granules and the ratio reaches very low levels when starch is gelatinised. When gelatinised samples are allowed to retrograde, the peak ratio begins to increase again and such a rise has been interpreted here as signifying the presence of retrograded material in mixtures used in the enzyme kinetic studies.

**Fig. 2:**
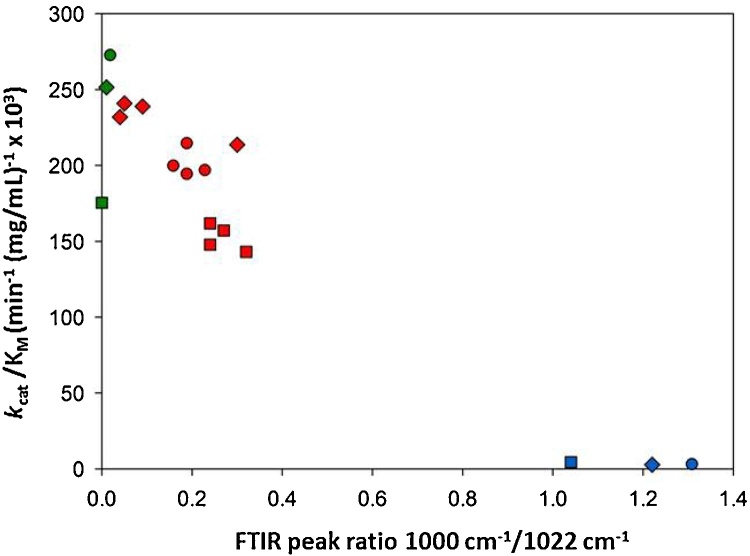
The relationship of catalytic efficiency (*k*_cat_/*K*_m_) with FTIR peak ratio for native, gelatinised and retrograded starch from potato (circles), wheat (squares) and pea (diamonds). The results obtained for native are shown in blue, gelatinised in green and retrograded in red. All experimental points are presented as mean values. (For interpretation of the references to colour in this figure legend, the reader is referred to the web version of this article.)

The relationship between the catalytic efficiency (CE) determined from measurements of *k*_cat_/*K*_m_ for wheat, pea and potato granular starches and their respective FTIR peak ratio is shown in Fig. 2. It is clear that the greater the proportion of disordered glucan chains, as assumed from the peak ratios, the greater is the CE. Native starches are normally highly ordered and have low CE values. The presence of retrograded starch is associated with a rise in peak ratio and this is accompanied by a fall in CE relative to the high values obtained for freshly gelatinised starches (i.e., containing no retrograded material). These results accord with previous findings that in the early stages of granule-PPA interaction, the enzyme binds to exposed surfaces of starch granules (Baldwin et al., 2015: Dhital, Gidley & Warren, 2015; Warren, Butterworth et al., 2013). For a number of different starches, catalytic binding as expressed by *K*_m_ values has been shown to be directly related to dissociation constants determined for amylase-granule binding under non-catalytic conditions (Warren, Butterworth et al., 2011). Thus productive binding seems to be limited only to sites where starch is susceptible to catalysed hydrolysis. Binding of the enzyme to flexible, relatively disordered, glucan chains and subsequent hydrolysis is a crucial step in the initial digestion of starch by PPA (Baldwin et al., 2015). Although the effects on PPA binding are likely to be the key reason for the fall in CE, if retrograded starch has a direct inhibitory action on PPA, this would lower *k*_cat_ and thus contribute to the observed decrease of CE. In our previous studies (Patel et al., 2014), a fall in the digestibility constant obtained from LOS plots and which is related to *k*_cat_ (Butterworth et al., 2012), could not be detected because of the excess of gelatinised starch that dominated the digestion kinetics. Testing for direct inhibition requires different kinetic strategies (see below).

**Fig. 3:**
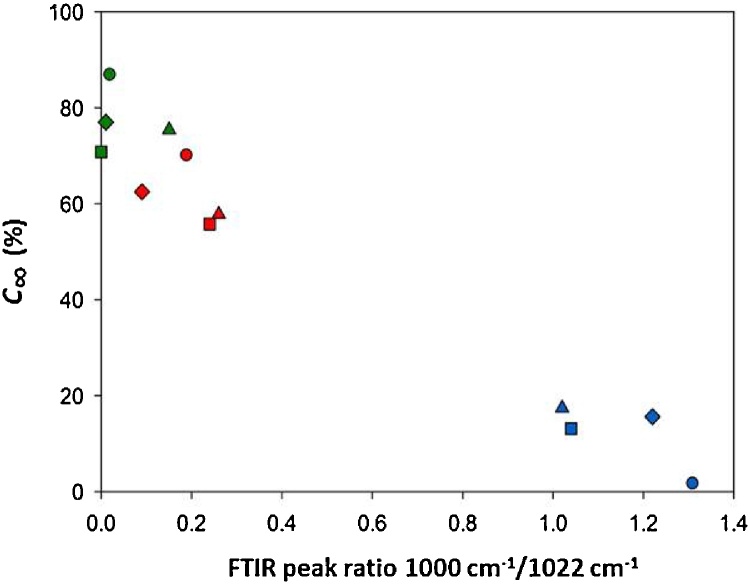
The relationship between the total digestible starch (C*_∞_*) and FTIR peak ratio for native, gelatinised and retrograded starch from potato (circles), wheat (squares) and pea (diamonds), maize (triangles). The results obtained for native starches are shown in blue, for gelatinised in green and retrograded in red. All experimental points are presented as mean values. (For interpretation of the references to colour in this figure legend, the reader is referred to the web version of this article.)

As expected, the total amount of digestible starch (C_∞_) obtained from LOS analysis of digestibility curves (Butterworth et al., 2012) also decreased with the rise in FTIR 1000 cm^−1^/1022 cm^−1^ peak ratio (Fig. 3). Native starches with relatively high peak ratios (i.e. highly ordered) had C_∞_ values that were <20% (range 2–17%) of the total starch present in digestibility mixtures but on gelatinisation the fall in peak ratio was accompanied by a marked increase in C_∞_ to 70–90%. (Examples of digestibility curves that were subjected to LOS analysis are shown in Fig.S1 of the Supporting Information). Fig. 3 also shows that for all the starches tested, allowing them to retrograde resulted in a decrease in C_∞_ commensurate with an increase in FTIR peak ratios relative to the results obtained for freshly gelatinised samples.

These results reveal that retrogradation of starch not only confers resistance to amylolysis, as evidenced by the decrease in C_∞_, but also impairs the catalytic efficiency of amylase. As mentioned above, a fall in CE can suggest that retrograded starch may act as an inhibitor of amylase and so this possibility was explored further by experiments using purified retrograded starch.

**Fig. 4:**
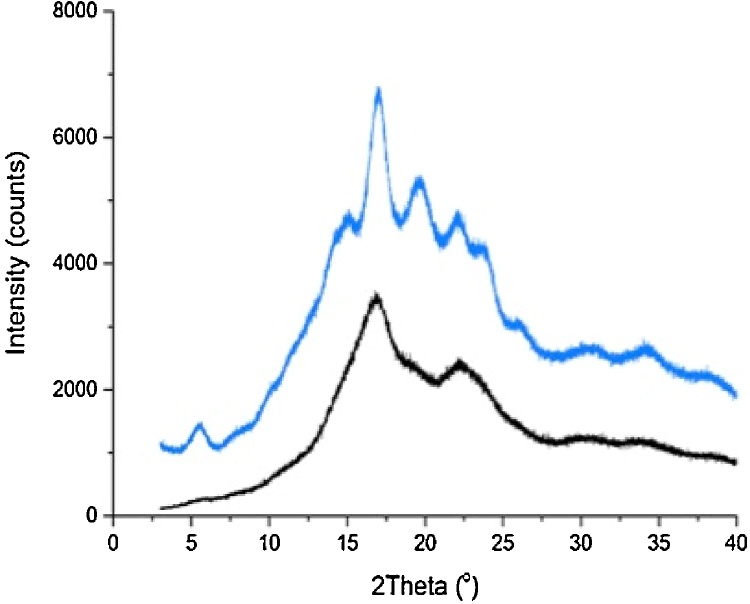
X-ray diffraction patterns of native HAMS (blue) and purified RHAMS (black). (For interpretation of the references to colour in this figure legend, the reader is referred to the web version of this article.)

RHAMS was characterised by X-ray diffraction (XRD), solid state NMR and DSC. Fig. 4 shows the XRD patterns obtained for native HAMS and RHAMS. Although some reflections commonly associated with B type crystallinity (Htoon et al., 2009) are missing from the RHAMS diffraction patterns, the reflections originally observed at 5° 2Ɵ and 14° 2Ɵ for native starch had disappeared and the double reflection between 22 and 25° 2Ɵ present in the native sample had combined into a single peak in the retrograded sample. The change in pattern is consistent with reports in the literature that the purified sample consisted of retrograded starch possessing a B-type crystallinity (Htoon et al., 2009; Sievert, Czuchajowska & Pomeranz, 1991; Gidley et al., 1995). For reliable estimates of the content of ordered material in the HAMS and RHAMS, samples were examined by ^13^C CP/MAS.NMR and ordered material found to be 33% and 50%, respectively. Thus the percentage of ordered material in the retrograded sample was considerably higher than that in the native HAMS. The presence of two peaks at 99 and 100 ppm in the C1 region of the spectrum reveal that the helical order is due to B-type starch (Gidley & Bociek, 1985) (Supporting information Fig.S2).

DSC gelatinisation parameters representing the melting of retrograded amylose in purified RHAMS were 125 ± 1.2 °C, 133.2 ± 3.2 °C and 141 ± 1.9 °C for T_o_, T_p_ and T_c_, respectively. The equivalent values of T_o_, T_p_ and T_c_ for native HAMS were 67.5 ± 1.7 °C, 87.5 ± 0.3 °C and 103.5 ± 0.9 °C (Supporting information Table S1). All the values are presented as the mean ± s.e.m. obtained from 3 replicates. Increases in gelatinisation temperatures accompanying retrogradation induced by temperature cycling have been reported previously (Sievert & Pomeranz, 1989).

Prior to its use in inhibition studies, RHAMS was incubated with PPA to check for any catalytic breakdown resulting from the presence of any non-retrograded starch remaining in the preparation. No detectable product was released during the initial rate studies.

**Fig. 5:**
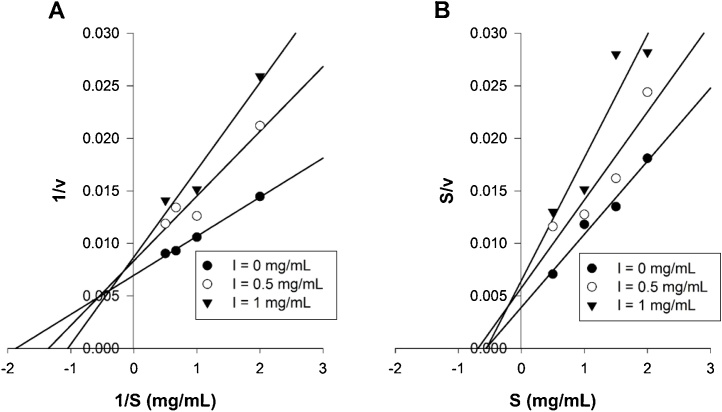
Inhibition of amylase activity acting on gelatinised wheat starch by RHAMS. In A the data are plotted in the form of 1/v against 1/S where v is the catalysed rate (mM/min) and S is the substrate concentration (mg/ml). In B the data are plotted in the form of S/v against S. Data points are means taken from 3 determinations each performed in duplicate.

Fig. 5 shows that RHAMS has a direct inhibitory action on the amylolysis of gelatinised wheat starch. The data were treated by using the familiar Lineweaver-Burk double reciprocal plot of 1/v against 1/S (Fig. 5A), where v and S represent the initial reaction velocity and starch concentration, respectively.

The plots of data at different concentrations of RHAMS were linear and convergent with intersections in the 4th quadrant. Such behaviour is indicative of a mixed pattern of inhibition (Cornish-Bowden, 2004). The plots were definitely not parallel. The same data are also plotted in Fig. 5B by the S/v against S method that is a recommended improvement on the double reciprocal plot (Cornish-Bowden, 2004). By the S/v *vs* S method, the plots were also convergent and indicative of mixed linear inhibition.

**Fig. 6:**
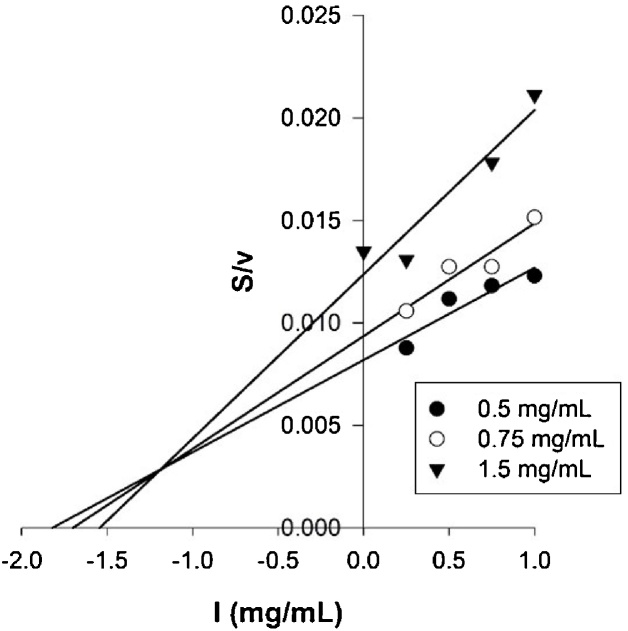
Cornish-Bowden S/v against I plots for action of amylase on gelatinised wheat starch in the presence of RHAMS. The concentrations of substrate are indicated in the inset. Data points are means taken from 3 determinations each performed in duplicate. The I value at the intersection point equals −*K_i_´*.

Cornish-Bowden plots of S/v against inhibitor concentration I are valuable for studying linear inhibition (Cornish-Bowden, 2004). The substrate concentration, S, was fixed at several different levels while RHAMS was included in a range of concentrations, I, and where v is the initial reaction velocity. Plots obtained for RHAMS are shown in Fig. 6.

The plots have a common point of intersection in the 4th quadrant, which can be indicative of either a mixed competitive-non-competitive pattern of inhibition or of an uncompetitive mode of inhibition (Cornish-Bowden, 2004). Uncompetitive inhibition results from an interaction of the inhibitor to the enzyme-substrate complex only, (i.e., binding to free enzyme does not occur) and can be identified from parallel double reciprocal plots. The intersection of the plots shown in Fig. 5 eliminates pure uncompetitive behaviour. A mixed pattern of inhibition implies that RHAMS can bind both to free PPA (E) and to the PPA-starch complex (ES).

The value of I at the point of intersection equals −*K_i_´*, the inhibition constant for I binding to the ES complex to form an ESI ternary complex, whereas the [S]/v value at the intersection point equals *K*_m_(1–*K_i_´*/*K*_i_)/V, where V is the maximum velocity of reaction and *K*_i_ is the inhibition constant for I binding to free enzyme to form an EI dead-end complex (Cornish-Bowden, 2004). From our data we estimated that *K*_i_ for our sample of RHAMS is approximately 0.5 mg/ml, whereas *K*_i_´ is approximately 1.2 mg/ml. We do not believe that these quoted values for the constants are important *per se*, but nevertheless feel that the kinetics provides the interesting result that binding to the free PPA is about twice as strong as binding to the ES complex.

Linear inhibition implies interaction at a single site on the enzyme, i.e., there is a 1:1 interaction of enzyme and RHAMS. The enzyme kinetics cannot provide direct information about the site on PPA to which RHAMS binds. X-ray crystallographic analysis of PPA and of human α-amylase, however, has identified the position of the catalytic site in the enzyme and suggests that the site binds five glucose residues for optimal activity but may also be able to accept a sixth glucose residue (Brayer et al., 2000; Gilles, Astier, Marchis-Mouren, Cambillau & Payan, 1996; Machius, Vertesy, Huber & Wiegand, 1996; Qian, Haser & Payan, 1994). Binding sites for other non-starch carbohydrates have also been identified on the surface of mammalian amylase (Gilles et al., 1996, Qian et al., 1994).

PPA binds to guar galactomannan which behaves as a non-competitive inhibitor (Slaughter, Ellis, Jackson & Butterworth, 2002) and recently, direct binding of PPA to cellulose was demonstrated with mixed linear inhibition of amylolysis (Dhital et al., 2015). The authors of the cellulose study concluded that binding of both cellulose and starch to the catalytic site region of PPA was unlikely because it is difficult to envisage how a molecule of PPA could be bound simultaneously to two very large molecules represented by starch and cellulose. The authors argue that cellulose could bind to a PPA-maltose end product complex without steric interference (Dhital et al., 2015). Such a mechanism would produce pure uncompetitive inhibition kinetics however (Byers, Fernley & Walker, 1972; Malik & Butterworth, 1977), rather than the mixed behaviour that we report for retrograded starch and which has been reported for cellulose. However, as the enzyme appears to be ‘sticky’ with respect to polysaccharides, once it became bound, amylase would be unable to interact with starch because of steric considerations. Preclusion of binding would be manifest in kinetic experiments as competitive inhibition. Thus retrograded starch and cellulose seem to exhibit mixed competitive-uncompetitive inhibition of α-amylase.

Interesting as the inhibition behaviour may be to enzymologists, the sticky property possessed by α-amylase has potential importance in a nutritional context. Direct inhibition of amylase by the presence in food of retrograded starch and/or dietary fibre can contribute to explanations for the observed effects on postprandial glycaemia associated with consumption of non-starch polysaccharide (NSP). For example, hitherto, the effects of water-soluble NSP (soluble fibre) on lowering glycaemic responses have been largely ascribed to increases in viscosity of intestinal contents. Such rheological changes in the gut lumen can attenuate a number of physiological processes including the rate of gastric emptying and affect the rate at which digestive enzymes interact with substrates in foods and also slow the rate of transfer of products away from the food-enzyme mix (Tharakan, Norman, Fryer & Bakalis, 2010). To this notion, a direct inhibition of α-amylase can now be added to any list of mechanisms proposed for explanation of the dietary actions of fibre. Applications arising from these results could perhaps lead to rational design of novel ingredients and foods that elicit a low rate and extent of starch digestibility and hence glucose absorption. These types of foods would have desirable health gains by reducing the risk of cardiometabolic conditions such as cardiovascular disease and type 2 diabetes.

## 4 Conclusions

Retrogradation of starch results in a fall in the total amount of starch that can be digested (C*_∞_*) and in the catalytic efficiency (CE) of PPA. The decrease in C*_∞_* is expected if retrograded starch (RS) is completely inert to amylolysis whereas a decrease in CE can reflect direct inhibition of PPA by RS.

Retrograded starch inhibited PPA by a mixed pattern of inhibition similar to that recently reported for cellulose inhibition. It seems that PPA can interact with various carbohydrate materials and that such a reaction inhibits catalytic activity. Therefore dietary consumption of retrograded starch may not only be beneficial to health through depletion of the total digestible starch, and therefore the metabolisable energy, but may also slow down the rate at which starch is digested in the intestine through direct inhibition of α-amylase. Such physiological effects have important implications for the prevention and management of type 2 diabetes.

## Appendix A Supplementary data

Table S1 contains gelatinisation parameters determined by DSC for the various starches. Fig. S1 shows amylase digestibility curves for native and retrograded starches from maize, waxy maize and high amylose maize, and Fig. S2 shows ^13^C CP/MAS.NMR spectra for high amylose starch in both native and retrograded state.
