# Supplementary material for: Structural and enzyme kinetic studies of retrograded starch: Inhibition of α-amylase and consequences for intestinal digestion of starch
Source: Carbohydr Polym. 2017 May 15;164:154–61. doi: 10.1016/j.carbpol.2017.01.040 (PMC5374268; doi:10.1016/j.carbpol.2017.01.040)
Supplement: Supplementary file 1 [file mmc1.docx]

**Supplementary data**

**Table S1**

Gelatinisation parameters determined by DSC for various starches. The data represent mean ± SEM values obtained from 3-4 experiments each performed in duplicate.

| Starch | Onset Temp  °C | Peak Temp  °C | Conclusion Temp  °C | Gelatinisation Range  °C | ΔH_gel_ (J/g) |
| --- | --- | --- | --- | --- | --- |
| Wheat | 47.8 ± 1.1 | 59.2 ± 0.2 | 74.1 ± 0.7 | 26.3 | 9.7 ± 0.9 |
| Potato | 59.5 ± 0.3 | 65.5 ± 0.0 | 70.3 ± 0.2 | 10.8 | 17.5 ± 0.8 |
| Pea | 48.0 ± 0.8 | 59.5 ± 0.0 | 77.3 ± 0.2 | 29.3 | 10.9 ± 0.5 |
| Maize | 59.9 ± 0.4 | 70.7 ± 0.2 | 79.2 ± 0.3 | 19.3 | 11.7 ± 0.6 |
| Waxy maize | 59.5 ± 1.4 | 73.0 ± 0.0 | 80.0 ± 0.3 | 20.5 | 10.2 ± 0.9 |
| HAMS* | 67.5 ± 1.7 | 87.5 ± 0.3 | 103.5 ± 0.9 | 36.0 | 5.5 ± 1.1 |

* High amylose maize starch.


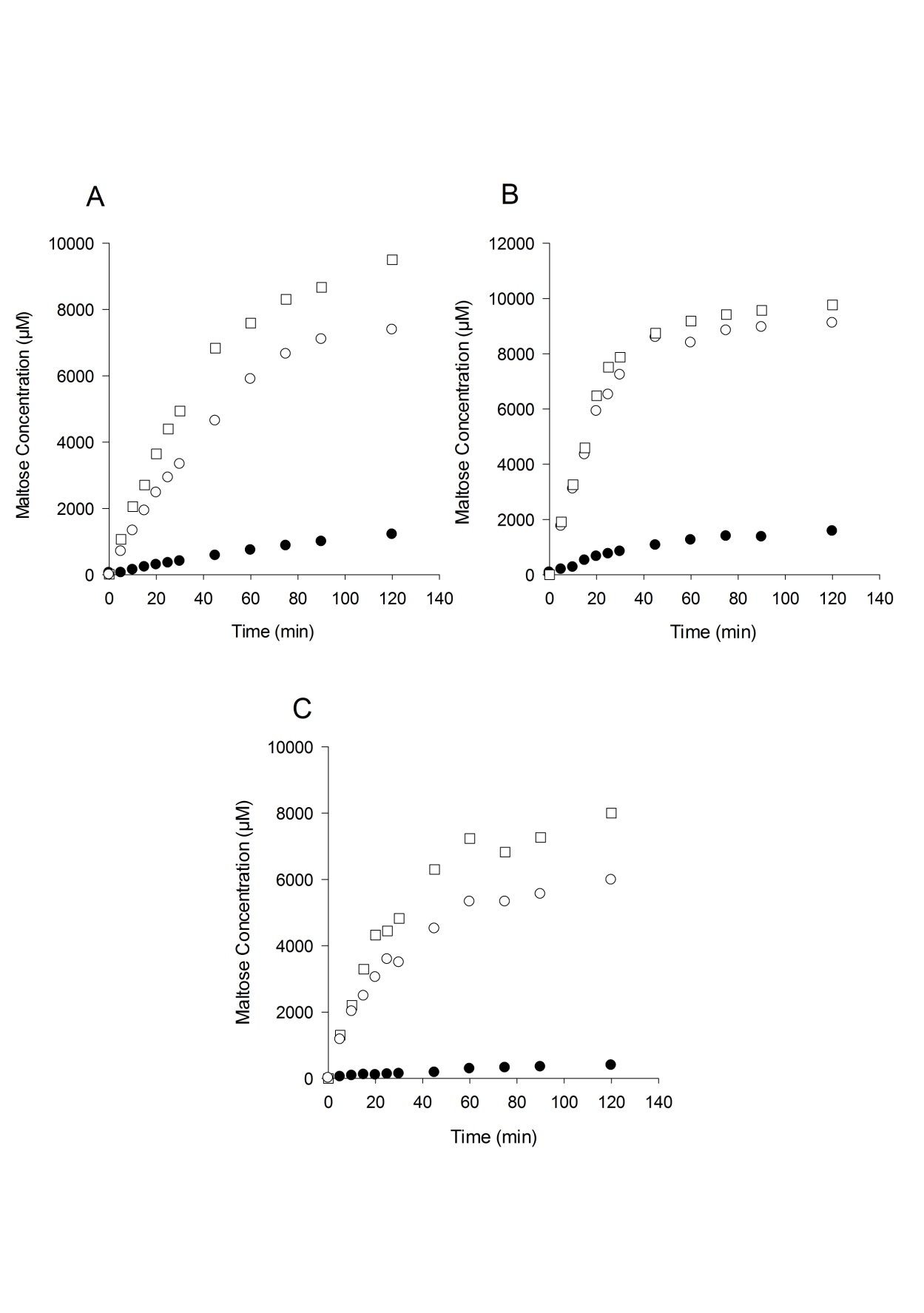


**Figure S1**. Digestibility curves of **native (●), gelatinised (🗆) and 24h retrograded (○) starches.** Maize (A), waxy maize (B) and high amylose maize starch (C). **Values for each data point are means obtained from three to four data sets.**

**Figure S2.** ^13^C CP/MAS.NMR spectra for HAMS and RHAMS
